# Using Digital Media to Empower Adolescents in Smoking Prevention: Mixed Methods Study

**DOI:** 10.2196/13031

**Published:** 2020-03-31

**Authors:** Eunhee Park, Yu-Ping Chang

**Affiliations:** 1 University at Buffalo Buffalo, NY United States

**Keywords:** adolescent, children, digital media production, technology, empowerment, smoking prevention

## Abstract

**Background:**

There is a critical need for effective health education methods for adolescent smoking prevention. The coproduction of antismoking videos shows promising results for adolescent health education.

**Objective:**

This study explored the feasibility of a smoking prevention program using the coproduction of antismoking videos in order to empower adolescents in smoking prevention and tobacco control. A smoking prevention program based on coproduction of antismoking videos over eight sessions was implemented in a low-income neighborhood.

**Methods:**

A mixed methods design with a concurrent embedded approach was used. In total, 23 adolescents participated in the program. During the prevention program, small groups of participants used video cameras and laptops to produce video clips containing antismoking messages. Quantitative data were analyzed using the Wilcoxon signed-rank test to examine changes in participants’ psychological empowerment levels between pre- and postintervention; qualitative interview data were analyzed using content analysis.

**Results:**

Pre- and postcomparison data revealed that participants’ psychological empowerment levels were significantly enhanced for all three domains—intrapersonal, interactional, and behavioral—of psychological empowerment (*P*<.05). Interviews confirmed that the coproduction of antismoking videos is feasible in empowering participants, by supporting nonsmoking behaviors and providing them with an opportunity to help build a smoke-free community.

**Conclusions:**

Both quantitative and qualitative data supported the feasibility of the coproduction of antismoking videos in empowering adolescents in smoking prevention. Coproduction of antismoking videos with adolescents was a beneficial health education method.

## Introduction

### Background

Preventing adolescents from becoming smokers is a crucial public health issue. Although the adolescent smoking rate has decreased overall, a significant majority (88%) of smokers have reported taking up the habit as adolescents [1]. Once having become an established smoker, quitting is difficult because of the addictive chemicals in tobacco products, often involving repeated attempts [1,2]. In addition, the adverse impacts of smoking can be more serious for adolescence-initiated long-term smokers because health outcomes last for the rest of their lives [3,4]. Therefore, it is important for prevention efforts to target adolescents who have not yet started smoking or who are just beginning to experiment with it [1,5]. Furthermore, adolescent smoking is particularly serious in minority populations residing in low-income neighborhoods [6,7]. However, prevention efforts have been challenging because this population is hard to reach, difficult to maintain engagement with, and typically suffers from a low level of health literacy and a chronic lack of resources [8,9]. Thus, it is vital to develop and provide socioculturally relevant interventions that take into account the characteristics of this population [10-13].

Engagement can be important for successful adolescent smoking prevention programs [14-16]. Engagement is defined as “the quality of effort students themselves devote to educational activities” [17] in an educational context. Learning outcomes are better achieved when youth are more engaged with pedagogically appropriate approaches [18-20]. There has been much effort focused on developing relevant content concerning adolescent smoking prevention; however, educational methods in delivering those topics have not been widely explored [4]. For example, researchers have suggested topics to be taught for smoking prevention, such as refusal skills, based on a cognitive behavioral approach [8,21,22]; these efforts have yielded some successful outcomes, but results have been mixed [1]. Thus, there is a critical need for more research on effective educational methods (eg, effective teaching methods and active learning methods) to better engage participants in the programs [14,15,23-25]. For adolescents, their developmental characteristics of seeking proper stimuli and fun activities with tasks containing level-appropriate challenges should be considered [20,26-28]. Technology and multimedia-related components are particularly useful to provide more engaging activities in this respect [27,29-31].

Empowerment is another important key that needs to be emphasized in smoking prevention programs in an effort to better help adolescents become advocates for tobacco control [32,33]. Youth empowerment approaches have been adopted for a number of adolescent health promotion interventions, including smoking prevention efforts, and have shown considerable promise [14,34-36]. This approach is known to be helpful in increasing adolescent engagement and in providing a voice, particularly for marginalized populations [37,38]. By adopting this approach, adolescents not only opt to resist smoking behaviors of their own volition but also become advocates for nonsmoking communities [34-37]. As smoking is a sociocritical issue, it is necessary to empower nonsmoking adolescents to become active advocates for a smoke-free society. This approach can be helpful to equip them with the information they need to understand smoking policy issues, how tobacco companies manipulate these issues, and how socioeconomic status influences smoking status.

Participatory media production or coproduction of films with participants has been used for educating adolescents about a number of social and health issues [39-42]. The coproduction process provides not only a method of engagement but also serves as a valuable tool to empower youth with regard to health and social issues [39,40,43-45]. In this study, we explored a newly developed adolescent smoking prevention program grounded in theory-based empowerment, using the coproduction of videos as a tool to facilitate adolescents’ engagement with self-expression and critical thinking. The purpose of this study was to examine whether coproduction of antismoking videos is feasible in empowering adolescent participants in smoking prevention.

### Theoretical Framework

This study was grounded in the Youth Empowerment Framework, adapted from Youth Empowerment Theory [46-48]. This framework was derived from the Nomological Network for Psychological Empowerment Model [49] and adapted for tobacco control. According to the Youth Empowerment Framework, psychological empowerment, which is rooted in social action theory, is defined as “empowerment at [the] individual level that integrates perceptions of personal control, a proactive approach to life, and a critical understanding of the sociopolitical environment” [50] and is composed of intrapersonal, interactional, and behavioral domains [47,48]. According to this framework, opportunities to gain control, mobilize resources, and critically understand sociopolitical issues enhance the psychological empowerment of young people [49]. The intrapersonal domain includes domain-specific efficacy, perceived sociopolitical control, and participatory competence; the interactional domain is composed of knowledge of resources, assertiveness, and advocacy; and the behavioral domain includes psychological empowerment-related actions (see Table MA1-1 in Multimedia Appendix 1) [50].

## Methods

### Study Design

The study protocol was approved by the affiliated Institutional Review Board, and consent from parents and minors was obtained. A mixed methods design with a concurrent, embedded experimental approach was used [51-53]. This mixed methods study concurrently collected both quantitative and qualitative data at the beginning and end of the intervention within the experimental design. However, the quantitative data from this concurrent, embedded experimental approach provided primary findings used to examine changes in primary outcomes before and after the intervention. The qualitative data were used to provide context and to support the quantitative findings. This study design is appropriate in the intervention test phase, as quantitative data will measure the primary outcomes of the intervention and qualitative data will provide participants’ feedback for an in-depth understanding of why and how those outcomes occur [53].

This design is suited to our objective of exploring the empowerment process involved with the intervention. Quantitative data from this study allow us to test the changes in psychological empowerment, which is the major outcome of this study. Additionally, qualitative data enable us to confirm whether findings from the quantitative analysis are related to the intervention and to explain how changes in the main outcome are related to components of the intervention. Quantitative data test the major hypothesis and the qualitative analysis provides additional insight and confirms findings based on quantitative data. Special care was taken to minimize potential threats to validity of the concurrent embedded design.

### Participants and Setting

This study was conducted in Pontiac, Michigan, USA, an urban, low-income neighborhood. The median household income was US $30,152 in 2012-2016, which is about half the US national average of US $55,322 [54]. The percentage of the population under the poverty level was 34.3%, about three times higher than the national average of 12.7%. Among those 25 years of age or older, 75.9% had a high school diploma or its equivalent, well below the US average of 85.2%. The unemployment rate in Pontiac was 10.90%, twice the national average of 5.20%. African Americans made up the single-largest ethnic group at 49.9%, well above the US average of 13.3%.

This study was conducted at a neighborhood, nonprofit community center in Pontiac that provides youth summer programs, and the intervention was embedded within their existing summer schedule. Participants who enrolled in the center’s summer program were approached for recruitment via flyers and handouts; 23 youths in grades 4-8 chose to participate in the study. The demographic survey indicated that 57% (13/23) of the participants were African American, 35% (8/23) were Latin American, and 9% (2/23) were Caucasian American. All the participants were nonsmokers, although 9% (2/23) had tried cigarettes before. The majority (15/23, 65%) were female and 35% were male (8/23). Gift cards valued at US $10 were given to the participants who completed the data collection.

### Intervention

The program consisted of eight sessions over a period of 4 weeks (see Table MA1-2 in Multimedia Appendix 1). Each session lasted 60 minutes. Groups of 3-5 students worked together to make a video clip over the course of the eight sessions. Instructors provided reading materials and access to websites for smoking-related content from reliable sources, such as the US Centers for Disease Control and Prevention, and each group of students discussed the determinants of smoking, the health consequences, and smoking prevention and cessation strategies. The instructors were experienced with children and were trained by the research team. Each group then picked their own topic and genre for their video clips. The resulting video clips lasted from 3 to 5 minutes, and every student had an opportunity to play a number of different roles, including camera person, actor, director, and/or writer. Based on the scenarios and storyboards they created, participants recorded different scenes using flip cameras and then edited the scenes using Windows Movie Maker 2014 (Microsoft). In the final session, they showed the video clips they had created to the teachers and other students at the center.

### Measures

Psychological empowerment is the major outcome of this study. The Youth Group Member Survey (YGMS) [47,48] was used to measure psychological empowerment for tobacco control. The YGMS was developed for adolescents, 10-21 years of age, and its validity and reliability have been supported with a Cronbach alpha of .86. The survey uses a Likert-type scale and consists of 19 items with three subscales: intrapersonal, interactional, and behavioral domains.

The intrapersonal domain considers an individual’s beliefs about their capacity to influence others’ lives, including their family, friends, surrounding environment, and sociopolitical context. This domain includes items such as “How sure are you that you can convince family members not to smoke?” The interactional domain refers to an individual’s understanding about the problems faced by their community and their assertiveness, and includes items such as “I can start discussions with others about tobacco issues.” The behavioral domain refers to an individual’s actions with empowerment, including nonsmoking intentions, advocacy actions toward a smoke-free community, and general actions that may influence other people’s lives. This domain includes items such as “Do you think you will smoke a cigarette at any time during the next year?” Quantitative measures only allowed us to assess their individual intentions to exhibit smoking behavior. Their advocacy actions or community actions were assessed via qualitative interviews.

### Data Collection

Quantitative data were collected using a pen-and-pencil survey at the beginning of the program and immediately after the program finished. Each survey lasted about 30 minutes. Immediately upon completion of the survey, a semistructured interview was conducted using an interview guide. The interview was conducted by a team member with previous experience interviewing children. The preintervention interview assessed adolescents’ motivation regarding the intervention, and the postintervention interview explored their experiences with the intervention process. Each interview lasted about 30 minutes.

### Data Analysis

A concurrent data analysis approach was used [52], with the quantitative and qualitative data being analyzed separately (Stage 1). The results were then compared for two datasets to explore whether and how the results supported each other (Stage 2) and were displayed jointly in the matrix using the theoretical framework (see Table 1). For the quantitative findings, we conducted a Wilcoxon signed-rank test as the normal distribution was not met, while the qualitative data was subjected to a content analysis based on the theoretical framework [55]. For the interviews, verbatim transcriptions were coded by two different coders for attributes (primary coding) and patterns (secondary coding). The primary coding followed an inductive approach, and the categories and themes that emerged were then organized using a deductive approach based on the theoretical framework in the secondary coding. Where there were discrepancies between coders, we discussed and resolved the disagreements in consultation with a third member of the research team. We utilized strategies to ensure trustworthiness [52].

## Results

### Overview

The statistical results from the pre- and postcomparison and representative quotes from the postinterviews that support the findings for each domain—intrapersonal, interactional, and behavioral—are presented in Table 1. The qualitative and quantitative data support each other well. In this study, all the domains of psychological empowerment were significantly enhanced, and the qualitative data facilitated the interpretation of these results by providing the context and meaning of these changes within this intervention. The narratives contributed to our understanding of the nature of the interventions. In particular, it was revealed that the participatory process, which was focused on the making of the health-related videos to show to others, lies at the heart of this intervention and was a key factor in the empowerment process for participants.

**Table 1:** Comparison of pre- and posttest results and example interview quotes for psychological empowerment.

Domain		Preintervention score, mean (SD)	Postintervention score, mean (SD)	*P* value	Example interview quotes
**Intrapersonal domain**		—^a^	—	—	“Smoking makes your teeth get yellow.” “I learned that smoking is bad for you.” “I just learned that smoking is bad and it could hurt your lungs and could affect your voice.” “Now I can actually tell people not to smoke.” “I can help other people not to smoke.” “I know not to smoke and to actually tell people not to smoke and stuff.” “I liked that I got to talk to [my friend] for her not to smoke just like in the video.”
	Perceived sociopolitical control	12.85 (2.37)	14.92 (2.37)	.01	—
	Participatory competence	7.23 (1.30)	9.00 (0.90)	.01	—
	Self-efficacy	10.18 (3.61)	14.79 (1.92)	.01	—
**Interactional domain**		—	—	—	“Really the only concern I have is that it might not affect some people the way that I think it will. That’s mainly my only concern I have.” “We’re making videos that helps people to stop smoking.” “I want to help and try to help any way I can though this program.”
	Advocacy	4.27 (2.91)	5.17 (2.92)	.01	—
	Assertiveness	10.55 (1.50)	13.60 (1.92)	.01	—
**Behavioral domain**		—	—	—	“This program has influenced me not to smoke.” “I am going to share this video to my friends and family, and some people who smoke.” “I would like to share. I have a cousin and I have friends that I still talk to. So I would definitely tell them about it and see if they can come or share the video that I make with them.” “...everybody in the world. I want to put this up on the YouTube.” “I like to do other videos too [about] substance use or drugs.” “I do community service with my school.”
	Smoking intention	12.91 (3.42)	14.70 (0.69)	.02	—

^a^Not applicable.

### Intrapersonal Domain

Our findings indicate that all subconstructs of the intrapersonal domain, including perceived sociopolitical control, participatory competence, and self-efficacy, were enhanced after the intervention (*P*<.05). By participating in the program, the youths showed enhanced self-competence and self-efficacy in remaining nonsmokers. Supported by the qualitative findings, most participants described how they had learned a great deal about the causes and consequences of smoking and had consequently become more confident in their ability to remain a nonsmoker. One participant stated, “Now I can actually tell people not to smoke,” in the postprogram interview. It was interesting to find that one of the main reasons given by many of the adolescents for participating in this study was that they were afraid they would start smoking due to influences from their surrounding environment. Participants also indicated that the video-making process helped them gain confidence in completing tasks and they expressed their pleasure in being the ones “in control,” unlike other programs that just “tell them what to do.” In addition, they showed self-efficacy in helping others not to smoke.

### Interactional Domain

In the interactional domain, advocacy and assertiveness also changed in a positive way (*P*<.05; see Table 1). Our qualitative findings showed that participants expressed a greater interest in and awareness of teenagers’ smoking issues and that they understood the complexity of the whole smoking issue, both of which helped them explore ways to help other people not to smoke. The participants suggested that making the videos could be a key way for them to engage in advocacy action, and they considered that this would be an effective way to persuade others. One participant stated:

We’re making videos that help people to stop smoking. Videos that will help people that might be going through something, they don’t know how to get out or to help somebody get out of a situation they were struggling in.

It was obvious that even after the program was finished, participants were thinking about ways to make the videos more effective. One participant stated:

Really the only concern I have is that it [the video produced] might not affect some people the way that I think it will. That’s mainly my only concern I have.

### Behavioral Domain

Positive changes were shown in participants’ intention to smoke in that they were less likely to initiate smoking after participating in this program (*P*<.05; see Table 1). Most participants strongly expressed their intention not to smoke in the future during the interview, as described by one participant: “This program has influenced me not to smoke.” Furthermore, they were interested in sharing the videos that they had made as an advocacy action for smoke-free communities. Participants wanted to share the videos with others, primarily their friends, family, relatives, and others they knew. They also wanted to show the videos they created to people beyond their extended social network and to make similar videos in the future to help other people not to smoke, as well as videos about other community issues. The intention to engage in empowered actions to help other people is clearly shown with video-making activities. This empowerment action was extended beyond video-making activities, as several of the participants indicated that they had started volunteering in their community, as shown by a participant’s statement: “I like to do other videos too [about] substance use or drugs.” Most of the participants wanted to become more actively involved in community issues, particularly with regard to drug use or violence-related issues, which indicates voluntary, empowered actions related to the surrounding community.

## Discussion

This study demonstrates how a participatory video approach, particularly using coproduction of antismoking videos, can empower adolescents in tobacco control. The study findings support the usefulness of technology in adolescent health education, particularly when exploring the video-making process for adolescent health education. Previously, technology was used as a delivery method and adolescents were treated as passive consumers of content, as digital media was emphasized as a final product delivered to adolescents in health education. This study advances the past approach by using coproduction of video clips for a smoking prevention program. The prevention program in this study allowed adolescent participants to be creative producers and active communicators using video cameras and laptops to create antismoking messages and to share those messages with others. In this way, technology was used for an active learning method in health education.

This study provides empirical evidence for the Youth Empowerment Framework [48]. In this study, the quantitative data showed statistically significant changes in all domains of psychological empowerment—intrapersonal, interactional, and behavioral—from before and after the smoking prevention program using an antismoking video-making process. In particular, the qualitative data confirmed that the adolescent participants experienced enhanced self-efficacy and competence for tobacco control, and their intention not to smoke was strengthened while participating in this smoking prevention program. In addition, the process of coproduction to create antismoking messages provided them with opportunities to become more aware of the resources available to them and the determinants of smoking. In the context of controlling tobacco use, the adolescents were able to identify ways to help others not to smoke and to make their community a smoke-free environment. Furthermore, the participants appreciated how this program allowed them to engage in advocacy actions for other smokers and teenagers and encouraged them to initiate further actions to help others in their communities, using both a digital media approach and other forms of voluntary activity. To our knowledge, this is the first study to explore the process of coproduction of antismoking videos for empowering adolescents in smoking prevention and the feasibility of the program utilizing a mixed methods approach.

However, there are limitations to note within the interpretation of the study findings. With convenience sampling, all the participants were recruited from a single site. This creates limitations concerning external validity, and the fact that it is based on the context of a summer camp in the United States needs to be noted in interpreting the findings. In addition, participants were a self-selected group, which introduced the selection bias of those who volunteered to participate in the study. They could have been highly motivated for smoking prevention and tobacco control. In addition, health literacy and reading skills of participants were not assessed, which could have influenced the findings of this study. Although the main purpose of this study was to test the feasibility of a newly developed prevention program using a coproduction approach, the lack of a control group limits our ability to confirm the effects of the program. We tried to minimize this limitation by using mixed methods so that both quantitative and qualitative data provided context and supported the outcomes of this intervention. In addition, though all levels of domain empowerment exhibited significant positive changes, we were not able to directly observe participants’ behaviors. The data sources of this study were from self-reported questionnaires and interviews; this may have introduced bias related to social desirability, meaning participants may have wished to please the investigators when completing the quantitative and qualitative data. Particularly, the behavioral domain was measured with one item that measured the intention to smoke, which does not provide information on advocacy actions or community actions; only qualitative data provide those aspects.

Future studies with a larger sample and a control group (eg, coproduction of other types of videos) are needed. It would also be helpful to explore the long-term effects, especially in studies that involve the direct observation of behavioral domain outcomes for empowered youth behavior and their impact as nonsmokers and advocates for a nonsmoking society, as well as their potential future as good citizens. With this methodological improvement, the efficacy or effect of coproduction on empowering participants in smoking prevention can be explored. Moreover, future studies can explore potential mechanisms by exploring any potential mediators or moderators. In addition, it will be interesting to explore whether the coproduction approach may influence other outcomes, such as critical thinking ability or leadership skills. Furthermore, it will be worthwhile to expand the components of coproducing the video clips to the generation and sharing of content by adolescents using two-way communication methods, such as social media for health education [31,56]. In addition, since e-cigarettes are an emerging issue for adolescents, developing an e-cigarette prevention program using participatory video production may need to be considered.

This study has major implications for health care practice and policy. Our findings suggest that coproduction of video clips about antismoking messages are able to empower adolescents to remain nonsmokers and become advocates for a smoke-free society in a low-income community. This study reports on a feasible way to use technology by incorporating appropriate pedagogical strategies for health education. With the process of coproduction of videos, participants may voice their opinions about specific social issues and become active participants for critical health issues. The findings suggest that the coproduction of videos was seen by participants as an opportunity to actively participate in social issues and help other people. The participatory video-making process may provide an example to help them understand health issues in depth. At the policy level, providing more resources or providing opportunities to better engage adolescents in smoking prevention programs could potentially have a significant impact, particularly for minority populations in low-income neighborhoods. The findings of this study suggest that coproduction of antismoking videos can be a useful and feasible educational method to engage this hard-to-reach population and empower these adolescents to be active participants in sociocritical health issues, such as tobacco control.

## Appendix

Multimedia Appendix 1 Supplementary tables.

## Abbreviations

YGMS: Youth Group Member Survey
