# Supplementary material for: Using Digital Media to Empower Adolescents in Smoking Prevention: Mixed Methods Study
Source: JMIR Pediatr Parent. 2020 Mar 31;3(1):e13031. doi: 10.2196/13031 (PMC7157503; doi:10.2196/13031)
Supplement: Multimedia Appendix 1 [file pediatrics_v3i1e13031_app1.docx]

**Table MA1-1. Operationalization of the Constructs of Psychological Empowerment**

| Domain | Description | General attributes | Attributes in tobacco control context | Definition (Holden et al., 2005) |
| --- | --- | --- | --- | --- |
| Intrapersonal | “Perceptions about themselves, including domain-specific perceived control; referring to beliefs about one’s ability to exert influence in different life spheres such as family, work, or sociopolitical context” (Zimmerman, 1995, p. 588) | Domain specific perceived control, self-efficacy, motivation to control, perceived competence. | Domain-specific efficacy | “Belief in one’s capacity to organize and execute the course of action required to produce specific changes related to tobacco control” (p.267) |
|  |  |  | Perceived sociopolitical control | “Beliefs about one’s capabilities and efficacy in social and political systems” (p.267) |
|  |  |  | Participatory competence | “Perceived ability to participate in and contribute to the operations of the group or organization, through talking at meetings, working as a team member, etc.” (p.267) |
| Interactional | “Understanding people have about their community and related sociopolitical issues. Awareness of behavioral options or choices to act”(Zimmerman, 1995, p. 589) | Critical awareness, understanding causal agents, skill development, skill transfer across life domains, and resource mobilization. | Knowledge of resources | “Awareness of whether resources exist to support the group and how to acquire them” (p.267) |
|  |  |  | Assertiveness | “Ability to express feelings, opinions, beliefs, and needs directly, openly, and honestly while not violating the personal rights of others” (p.267) |
|  |  |  | Advocacy | “Pursuit of influencing outcomes, including public policy and resource allocation decisions within political, economic, and social systems and institutions that directly affect people’s lives” (p.267) |
| Behavior | Actions taken directly influence outcomes, such as engaging in actions to change one’s community | Community involvement, organization participation, coping behaviors | Non-smoking | Behavioral intentions for non-smoking |
|  |  |  | Advocacy actions for smoke-free community | Behavioral intentions for community actions for smoke-free community |

**Table MA1-2. Description of Intervention Sessions**

| Phase | Weeks | Session | Program Activities | Smoking-related content |
| --- | --- | --- | --- | --- |
| Pre-production | Week 1 | Session 1 | Ice breaking, introducing each other, making a group, goal sharing, setting rules, ethical training | Consequences of smoking, occurrence of teenager smoking |
|  |  | Session 2 | Interviewing activities, identifying issues, setting audiences, and planning the message of video products | Different factors related to smoking, including peer-influence, media, policy, and tobacco companies. |
|  | Week 2 | Session 3 | Planning the scenes and plots, writing the scripts | Strategies for smoking prevention and cessation |
| Production |  | Session 4 | Filming | Topics selected by each group  Group 1) Situations of adolescents’ smoking initiation and consequences  Group 2) Consequences for young smokers  Group 3) Harmfulness of smoking  Group 4) Factors related to adolescent smoking (academic stress and peer pressure), health consequences, and coping strategies |
|  | Week 3 | Session 5 | Filming and editing |  |
|  |  | Session 6 | Editing |  |
| Post-production | Week 4 | Session 7 | Evaluating |  |
|  |  | Session 8 | Community forum and reflection |  |
